# Natural History of Treated and Untreated Bland Portal Vein Thrombosis in Patients with Hepatocellular Carcinoma

**DOI:** 10.3390/cancers18132148

**Published:** 2026-07-03

**Authors:** Tim Weber, Antonina Antonenko, Jonas Schropp, Pompilia Radu, Annalisa Berzigotti

**Affiliations:** Department of Visceral Surgery and Medicine, Inselspital, Bern University Hospital, University of Bern, 3010 Bern, Switzerland

**Keywords:** cirrhosis, thrombosis, portal hypertension

## Abstract

**Background/Objectives**: The prevalence and management options of bland (non-neoplastic) portal vein thrombosis (PVT) in patients with hepatocellular carcinoma (HCC) are poorly characterized and data remain limited. **Methods**: We performed a retrospective analysis of a prospectively collected single-center cohort of 638 patients with HCC. Bland PVT was identified at baseline or during follow-up. Treatment exposure was modeled as time-varying to account for differences in timing of anticoagulation initiation. **Results**: Bland PVT was identified in 39 patients (25 at baseline and 14 during follow-up). Patients with PVT showed features of more advanced portal hypertension (lower platelet count and higher prevalence of ascites) and had a higher tumor burden (more often outside the Milan criteria; higher AFP). A total of 30 patients received anticoagulation. Anticoagulation was associated with partial or complete recanalization in 30% of cases at three months and with decrease in bilirubin, but was not associated with improved 1 year survival. Bleeding events occurred in four anticoagulated patients (13.3%). **Conclusions**: Bland PVT in HCC is associated with more severe liver disease. Our data indicate that anticoagulation in patients with HCC and concomitant bland PVT was generally well-tolerated and was associated with thrombus regression in one-third of cases. However, we did not observe an improved survival in patients on anticoagulation.

## Simple Summary

A blood clot in the main vein supplying the liver (called the portal vein) can occur in people with liver cancer and liver cirrhosis. When this clot is not caused by the cancer itself, it is known as a bland clot. However, little is known about how often these clots occur or how they should be treated. In this study, we followed 638 patients with liver cancer and found that bland portal vein clots were relatively uncommon. They occurred more often in patients with more advanced liver disease and signs of increased pressure in the portal vein. Most patients with these clots received blood-thinning medication (anticoagulation). About one in three showed partial or complete improvement of the clot after three months, and liver function also improved in some patients. Although anticoagulation did not increase survival, it was generally well tolerated, with relatively few serious bleeding complications, suggesting it can be safely considered in carefully selected patients..

## 1. Introduction

PVT is a common complication in patients with cirrhosis, with a prevalence ranging from 3.7% to 24.4%; patients with decompensated disease have the highest prevalence and incidence [1,2]. The risk of PVT is even higher in patients with HCC, which can be seen as a prothrombotic condition [3]. In this population, not unexpectedly, PVT seems to worsen outcomes irrespective of tumor-related factors and limit the therapeutic options for HCC [4,5]. Anticoagulation has been shown to be effective in achieving recanalization and avoiding worsening of PVT, as well as improving clinical outcomes in patients with cirrhosis and no HCC [6,7]. However, the evidence regarding safety and efficacy of anticoagulation in patients with bland PVT and concomitant HCC is very limited and conflicting, with some studies suggesting improved outcomes in treated patients [5,6], while others showing no difference in recanalization rates with or without anticoagulation [8,9,10,11,12].

Given the scarcity of data regarding the natural history of treated and untreated bland PVT in the context of HCC, we aimed to review our own experience in this setting.

## 2. Materials and Methods

In this single-center retrospective study, we reviewed the clinical, laboratory and imaging data of patients with bland PVT in the prospective cohort of patients with HCC treated and followed-up at the Inselspital, University Hospital of Bern (Switzerland). All subjects in the study were adults (aged ≥ 18 years) prospectively enrolled between 11 August 2010 and 10 October 2023, who had signed the general consent for the use of their health-related data. The diagnosis of HCC was based on EASL guidelines [13]. All patients underwent radiological follow-up every 3 months. Splanchnic vessel patency, HCC characteristics, demographic, clinical and laboratory data were collected from medical records at enrolment, 3 months, 6 months and 12 months of follow-up. Causes of death were derived from medical records and classified as HCC-related (liver-related death in which there was evidence of progression or recurrence of the neoplastic disease) or HCC-unrelated. The radiological images were evaluated by radiologists with specific expertise in cirrhosis and HCC, and in case of insufficiently described nature of PVT in the radiology report, they were re-evaluated by an internal expert to distinguish between bland and neoplastic thrombosis. Suspicion and diagnosis of neoplastic thrombosis were established based on the following radiological criteria: the close proximity of the tumor to the vessel, the continuity of the thrombus with the HCC nodules, the thrombus expanding the vessel lumen to over 23 mm in diameter, and the presence of wash-in in the arterial phase [14].

The percentage of vessel occlusion in each splanchnic venous segment (main portal vein, intrahepatic portal branches, superior mesenteric vein and splenic vein) was assessed on the portal venous phase on contrast-enhanced images. According to the latest expert guidelines, PVT was classified as minimally occlusive if the clot obstructed < 50% of the lumen, partially occlusive when the clot obstructed more than 50% of the original vessel lumen, and as completely occlusive when no residual lumen was present [15].

This study was approved by the Cantonal Ethical Committee of Bern (Number 2023-01719; date 13 November 2023) and the study protocol conformed to the ethical guidelines of the 1975 Declaration of Helsinki.

### Statistical Analysis

Continuous variables were summarized using appropriate measures of central tendency and dispersion depending on their distribution. Group comparisons between patients with and without anticoagulation therapy were performed using Student’s *t*-test or the Mann–Whitney U test, as appropriate. Qualitative data are described using frequencies and percentages and compared using the χ^2^ test or Fisher’s exact test. Survival analysis was performed using the Kaplan–Meier estimator. As anticoagulation could be initiated at different time points during follow-up, treatment status was modeled as a time-varying exposure, allowing patients to enter the risk set accordingly. Survival curves therefore reflect this time-dependent treatment allocation. Given the potential for non-proportional hazards, differences between groups were evaluated using the supremum test. Patients were censored at the date they were last seen at the clinic or at closure of data collection to mitigate immortal time bias.

The longitudinal analyses were performed using generalized linear mixed-effects models (GLMMs) with random intercepts for each patient to account for repeated measurements within patients over time (baseline, 3, 6, and 12 months). Fixed effects included group (anticoagulation vs. no anticoagulation), time, and their interaction. Depending on the distribution of each outcome, appropriate model specifications were applied: approximately normally distributed variables (e.g., cholesterol, albumin, INR) were analyzed using Gaussian models, skewed variables (bilirubin and AFP) using log-normal or Gamma models with results presented as geometric means on the original scale, and ordinal outcomes (MELD score and AKI stage) using cumulative link (ordinal) models with results summarized as medians based on predicted probabilities. Model-based marginal estimates with 95% confidence intervals or medians with interquartile ranges at baseline, 3, 6, and 12 months are reported. Statistical inference was based on Type III Wald χ^2^ tests for the main effects of group, time, and their interaction. Because patients were followed up every three months, the effect of anticoagulation therapy on ascites was calculated using discrete time survival models implemented as generalized linear models with complementary log-log link and an offset for interval duration, with results expressed as hazard ratios (HRs) and 95% confidence intervals.

All analyses were done with case-wise exclusion of missing data. A *p*-value of <0.05 was considered statistically significant.

Generative artificial intelligence has not been used in this paper, except for polishing the English language.

## 3. Results

The cohort consisted of 638 patients with HCC. Bland PVT was diagnosed in 25 patients (3.9%) at baseline (first HCC diagnosis). During the follow-up, an additional 14 patients initially showing a patent portal venous system developed PVT (Table 1). All patients were followed for 12 months after the diagnosis was established. Tumoral invasion of portal venous vessels occurred much more frequently, being seen in 79 (12.4%) patients in total.

**Figure 1:**
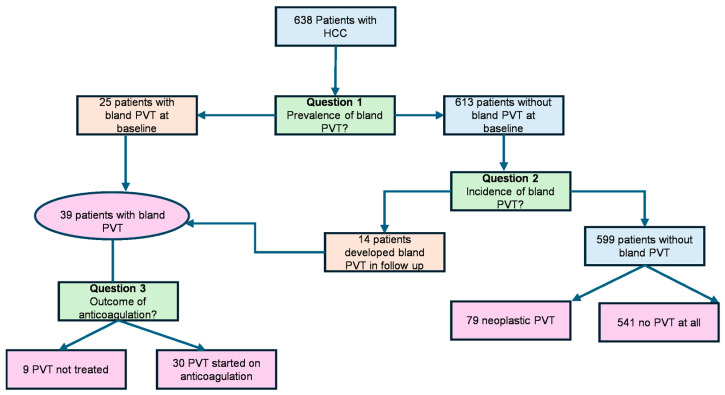
Study flowchart illustrating the prevalence of portal vein thrombosis (PVT) in patients with hepatocellular carcinoma (HCC) at baseline and during follow-up.

The flowchart (Figure 1) illustrates our study of PVT in 638 patients with HCC and provides a structured overview of PVT occurrence and management in this patient cohort.

We compared the baseline characteristics of patients with and without bland PVT. Age, MELD score and serum albumin are similar between the groups. Ascites was more frequent among PVT patients compared to non-PVT patients (30.1% vs. 11.2%; *p* = 0.008), and patients with PVT had significantly lower platelet count (119 ± 59 G/L) compared to those without PVT (162 ± 84 G/L; *p* = 0.002). As shown in Supplementary Figure S1, patients who developed PVT during follow-up had significantly lower baseline platelet counts (93.9 ± 26.5 G/L) compared with those who did not develop PVT (163.5 ± 83.4 G/L; *p* = 0.0003). Patients with bland PVT showed larger nodules as compared to those without PVT—(53.6 ± 46.7 mm) vs. (42.5 ± 36.4 mm)—although this difference did not reach statistical significance (*p* = 0.09). Alpha-fetoprotein (AFP) levels were significantly higher in patients with PVT (5372.5 ± 22,218.7 ng/mL) compared with those without PVT (795.4 ± 6857.2 ng/mL; *p* = 0.002). Additionally, a significantly larger proportion of PVT patients fell outside the Milan Criteria for liver transplantation (71.8%) compared to non-PVT patients (46.6%; *p* = 0.003).

### 3.1. Outcomes in Patients with Bland PVT Receiving vs. Not Receiving Anticoagulation

Among the 39 patients diagnosed with bland thrombosis, 30 (76.9%) received anticoagulation, while 9 (23.1%) did not (Table 2).

As for the type of anticoagulants, rivaroxaban was prescribed in 17 cases, apixaban in eight cases, and low-molecular-weight heparin (LMWH) in five cases.

The reason why anticoagulants were not started in the nine untreated patients varied (thrombus was considered too small to warrant treatment; watch-and-wait approach; presence of anemia or an elevated perioperative bleeding risk, particularly in patients scheduled for orthotopic liver transplantation or transarterial embolization; and systemic therapy alone was considered necessary and sufficient).

Baseline characteristics such as age, body mass index (BMI), bilirubin, sodium, platelet count, AFP, HbA1c, and albumin did not differ significantly between the two groups, indicating a comparable clinical profile. After three months, complete or partial recanalization was documented in 9 of 30 anticoagulated patients (30.0%) and in 2 of 9 untreated patients (22.2%).

Three of the four bleedings occurred within the first three months of therapy. Bleedings were attributed to portal hypertension (gastroesophageal variceal bleeding; portal hypertensive gastropathy; rectal varices). Within the non-anticoagulated cohort, no bleeding complications were documented.

### 3.2. Changes in Liver Function in Patients with and Without Anticoagulation

In the follow-up, most parameters evolved similarly in patients with and without anticoagulation (Table 3).

While bilirubin values were significantly lower during the follow-up in patients receiving anticoagulants (*p* = 0.02), no benefit was observed for albumin, INR, MELD score, AFP, or platelets. Importantly, the interaction analysis over time did not reveal sustained or uniform improvements across multiple laboratory values.

### 3.3. Survival After Portal Vein Thrombosis: Anticoagulation Versus No Anticoagulation

**Figure 2:**
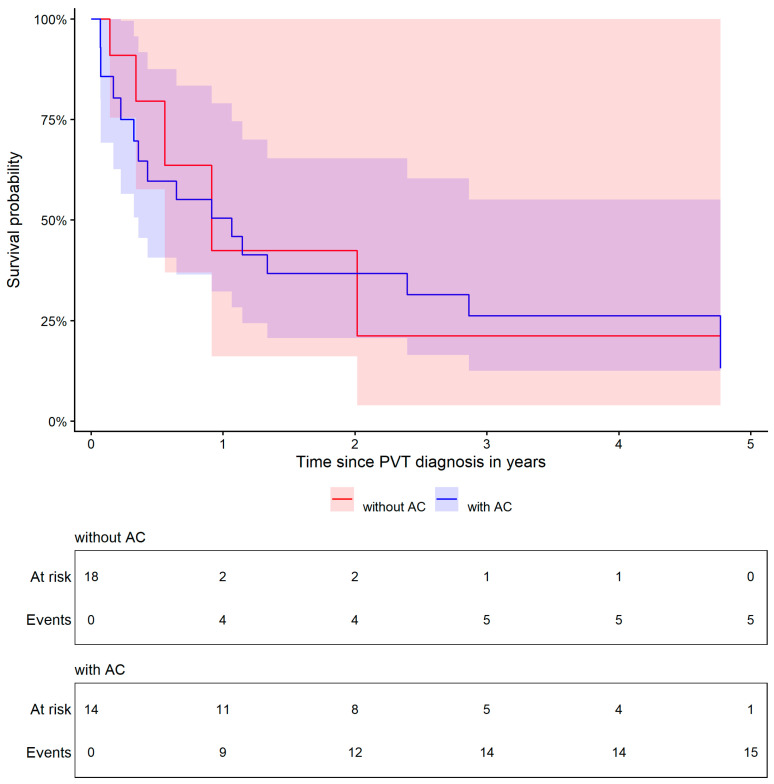
Kaplan–Meier estimates of survival probabilities according to the presence of anticoagulation therapy and its evolution pattern. Abbreviations: PVT—bland portal vein thrombosis; AC—anticoagulation.

A time-dependent Kaplan–Meier survival analysis was performed to visualize the survival probability between patients receiving anticoagulation therapy and those who did not, following a diagnosis of PVT (Figure 2).

Because anticoagulation therapy could be initiated any time during the follow up, a time-dependent analysis in all patients with available treatment dates was conducted. As shown, patients receiving anticoagulation therapy (blue line) did not demonstrate a higher survival probability over time compared to those without anticoagulation (red line). Due to the time-varying treatment initiation and potential for non-proportional hazards, the supremum test was used to statistically assess differences in survival distributions between the groups. The analysis revealed no significant difference (*p*-value = 0.657), showing no statistical support that anticoagulation therapy is associated with survival outcomes in patients with PVT.

## 4. Discussion

Solid and hematological cancers are often associated with a paraneoplastic prothrombotic syndrome [16]. Venous thromboembolic disease related to cancer is linked to reduced survival, increased morbidity, and higher hospitalization rates [17].

In patients with cirrhosis and HCC, bland PVT may represent a clinically relevant manifestation of the prothrombotic state associated with both advanced liver disease and cancer. Given that PVT may further worsen portal hypertension, which is, per se, an aggravating factor of prognosis in patients with HCC, more data on the prevalence, incidence, natural history and on how to treat this complication in the setting of HCC are needed.

In our study, we identified bland PVT in 3.9% of cases at baseline. Furthermore, 2.2% of patients who initially did not present with PVT developed it during the follow-up. This underlines that bland PVT is not rare in patients with HCC, and should be closely monitored during the follow-up.

Interestingly, while liver function was similar between patients with and without PVT, ascites and a lower platelet count were associated with PVT, suggesting worse portal hypertensive status as compared to patients who did not have and did not develop PVT.

The prevalence of bland PVT observed in our cohort is consistent with previous reports in patients with HCC, although direct comparisons are hampered by heterogeneous diagnostic criteria and follow-up durations across studies. In the largest comparative series to date, Benevento et al. [10] reported similar Child–Pugh and MELD scores between HCC and non-HCC patients with PVT, as well as comparable degrees of main portal vein involvement, suggesting that the underlying severity of portal hypertension, rather than the presence of HCC itself, is the principal driver of thrombus extension. Our findings are in line with this concept: patients with bland PVT in our cohort displayed lower platelet counts and a higher prevalence of ascites compared with those without PVT, both surrogate markers of more advanced portal hypertension, while liver synthetic function (bilirubin, INR, albumin) did not differ significantly between groups. This pattern, also described by Senzolo et al. [9] in their prospective cohort of patients with cirrhosis and newly diagnosed HCC, supports the notion that bland PVT in this population arises predominantly from hemodynamic rather than purely oncologic mechanisms, even though elevated AFP levels in our PVT cohort point toward a parallel contribution of overall tumor burden.

The majority of patients with bland PVT at our center study were started on anticoagulation. Anticoagulants in patients with PVT and cirrhosis but no HCC have shown beneficial effects beyond thrombosis recanalization, including a benefit in survival. This effect is attributed to the improved sinusoidal microcirculation leading to an improvement in liver function [18,19]. A recently published randomized controlled trial further supports this concept, showing that rivaroxaban reduced portal hypertension-related complications and tended to improve clinical outcomes in patients with cirrhosis [18]. In our treated patients we did not observe changes in liver function after starting anticoagulation. We observed that one-third of patients experienced partial or complete recanalization of thrombosis while on anticoagulation therapy. This was found to occur at a lower level in untreated cases (22%).

The recanalization rate observed in our anticoagulated patients (30% at three months) falls within the range reported in mixed cirrhotic populations with and without HCC, although recanalization rates across studies vary considerably depending on the degree of baseline vessel occlusion, a variable that our study collected but did not formally incorporate into outcome models due to sample size constraints. The extent of thrombus occlusion at baseline is a recognized determinant of recanalization in non-HCC cirrhotic populations, with minimally occlusive thrombi showing substantially higher spontaneous and treatment-associated resolution rates than partially or completely occlusive ones [12]. Given that our cohort included patients across the full spectrum of occlusion grades, this heterogeneity likely contributed to the modest overall recanalization rate and should be considered when comparing our results with studies enrolling more homogeneous populations in terms of thrombus extent.

Regarding liver function, anticoagulation in our series was associated with a reduction in bilirubin but not with sustained improvement in albumin, INR, MELD score, or platelet count. This contrasts with data from non-HCC cirrhotic populations, where anticoagulation has been linked to broader improvements in hepatic synthetic function, attributed to enhanced sinusoidal microcirculation following recanalization [6,7], a concept further supported by the recent CIRROXABAN trial showing that rivaroxaban reduced portal hypertension-related complications in patients with cirrhosis independent of overt PVT [20]. The more limited improvement observed in our HCC population may reflect the competing influence of active tumor burden on liver function trajectories, which could offset or mask the hemodynamic benefit of anticoagulation seen in non-malignant settings. Additionally, in our cohort, patients selected for anticoagulation had lower baseline platelet counts than untreated patients, indicating more advanced portal hypertension in the treated group; this imbalance, while not statistically significant, represents a plausible confounder that may have attenuated any detectable benefit of anticoagulation on liver function and warrants consideration in future prospective studies with larger sample sizes.

Portal hypertension itself increases the likelihood of developing PVT in cirrhosis, and could be an important confounder of the relationship between anticoagulation and bleeding risk. In our study population, all bleeding episodes among anticoagulated patients originated from portal hypertensive sources, underscoring the need for early and appropriate management of portal hypertension such as the initiation of non-selective beta-blockers to reduce the risk of bleeding, as suggested by the current guidelines on PVT in cirrhosis [21].

Despite the favorable signal on thrombus recanalization, anticoagulation was not associated with improved one-year survival in our cohort. This finding diverges from data in non-HCC cirrhotic populations, where anticoagulation-associated recanalization has been linked to survival benefit [5,6], and partially from a subset of HCC-specific studies suggesting improved outcomes in treated patients [8]. However, other series specifically enrolling patients with concomitant HCC have failed to demonstrate a consistent survival advantage with anticoagulation [9,10,11], mirroring our results. We hypothesize that in the HCC setting, overall survival is predominantly determined by tumor stage, treatment eligibility, and response to oncologic therapy rather than by portal venous patency alone, particularly in a cohort such as ours where a markedly higher proportion of PVT patients fell outside Milan criteria (71.8% vs. 46.6% in patients without PVT, *p* = 0.003). This observation reinforces the concept that bland PVT in HCC, while treatable, may primarily serve as a marker of more advanced underlying liver and tumor disease rather than an independent modifiable prognostic factor, a distinction that has direct implications for how anticoagulation should be framed to patients and clinicians—as a strategy to preserve vascular patency and treatment eligibility (e.g., for liver transplantation or locoregional therapies), rather than as a survival-prolonging intervention per se.

Our study has several limitations that are inherent to its retrospective nature. The number of cases is not large, and, in particular, the number of untreated PVT is small, and due to this, the results regarding recanalization of PVT and survival probability have to be interpreted with caution. The differential diagnosis between bland and neoplastic PVT has been in some cases challenging and a definitive diagnosis would require a fine-needle biopsy of the thrombus, which is rarely performed due to the potential risks and technical complexity. The recent EASL Clinical Practice Guidelines on vascular diseases of the liver [21] and the Baveno VIII consensus accept a differentiation between bland and neoplastic thrombosis based on the best available non-invasive diagnostic criteria, since contrast-enhanced imaging modalities have demonstrated high diagnostic accuracy for distinguishing between the two, with reported sensitivity and specificity generally exceeding 90% [22]. Despite our best efforts to ensure an accurate interpretation of patients’ images, we cannot fully exclude the possibility that some patients diagnosed with bland PVT may actually have HCC-related vascular invasion, which would not be responsive to anticoagulation.

## 5. Conclusions

In conclusion, our findings show that anticoagulation can lead to partial or complete recanalization of bland PVT in patients with HCC, but is not clearly associated with survival benefit. Further studies, including randomized controlled trials, are necessary to confirm these findings and optimize anticoagulation strategies in this patient population.

## Figures and Tables

**Table 1:** Baseline characteristics of patients with hepatocellular carcinoma (HCC) with and without benign portal vein thrombosis (PVT) at baseline.

	PVT at Baseline— N = 25	No PVT— N = 613	*p*-Value
Age, y	65.4 (±9)	65.5 (±9.8)	0.926
Sex—Female, n (%)	8 (20.5%)	94 (15.7%)	0.497
BMI, kg/m^2^	27.9 (±4.7)	27.1 (±4.7)	0.321
***Liver disease and HCC***			
Creatinine (mg/dL)	0.9 (±0.263)	0.86 (±0.49)	0.617
Bilirubin (mg/dL)	1.23 (±0.723)	1.23 (±2.376)	0.991
INR	1.21 (±0.22)	1.18 (±0.26)	0.391
Albumin (g/L)	31.6 (±6.9)	33.3 (±5.75)	0.74
Platelets (G/L)	118.8 (±58.9)	161.7 (±83.6)	0.002
Sodium (mmol/L)	138.9 (±3.4)	139.1 (±3.3)	0.702
Etiology of liver disease, n (%)			
Alcohol-induced LD	16 (41)	262 (45)	0.632
MASH	14 (35)	181 (31.1)	0.594
HCV	9 (23)	164 (28.4)	0.582
HBV	6 (15.3)	128 (22.1)	0.423
HIV, n (%)	2 (0.5)	5 (0.9)	0.072
Cirrhosis, n (%)	34 (87.1)	453 (77.7)	0.227
MELD score	7.35 (±4.7)	6.6 (±5.1)	0.374
ALBI score (≤−2.60/−2.60) <ALBI score ≤−1.39/>−1.39, n (%)	−1.85 (+/−0.656)	−1.91 (+/−0.837)	0.703
Ascites, n (%)	12 (30.1)	78 (11.2)	0.008
Encephalopathy, n (%)	2 (5.1)	20 (2.9)	0.644
Number of nodules	7.2 (±22.1)	2.2 (±1.6)	0.170
Size of nodules (mm)	53.6 (±46.7)	42.5 (±36.4)	0.09
Not within Milan Criteria, n (%)	28 (71.8)	264 (46.6)	0.003
BCLC			0.225
0	3 (7.7)	42 (6)	
A	15 (38.4)	254 (36.5)	
B	6 (15.4)	165 (23.7)	
C	12 (30.7)	91 (13.1)	
D	2 (5.1)	20 (2.8)	
Extrahepatic metastases, n (%)	6 (15.4)	45 (8)	0.100
AFP	5372 (±22,219)	795 (±6857)	0.002
***Comorbidities and dependencies***			
Smoking (PY)	39.2 (±25)	33.7 (±21.2)	0.361
Smoking status			0.828
Never smoker	20 (51.3)	271 (47)	
Current smoker	11 (28.2)	164 (28.5)	
Former smoker	8 (20.5)	141 (24.5)	
Alcohol consumption years (>30 mg/d)	24.7 (±15.5)	26.2 (±13.6)	0.696
Diabetes mellitus, n (%)	14 (35.9)	198 (34.4)	0.863
Arterial hypertension, n (%)	17 (43.6)	155 (58%)	0.119
Coronary heart disease, n (%)	5 (12.8)	81 (14.1)	1.000
***Performance status***			
ECOG, n (%)			0.343
0	28 (71.8)	367 (53)	
1	8 (20.5)	150 (21.6)	
2	1 (2.5)	43 (6.2)	
3	1 (2.5)	12 (1.7)	
4	3 (7.7)	1 (0.1)	

Abbreviations: AFP, alpha-fetoprotein; ALBI, albumin–bilirubin score; BCLC, Barcelona Clinic Liver Cancer; BMI, body mass index; HBV, hepatitis B virus; HCC, hepatocellular carcinoma; HCV, hepatitis C virus; INR, international normalized ratio; MELD, Model for End-Stage Liver Disease; MASH, metabolic dysfunction–associated steatohepatitis; PVT, portal vein thrombosis; PY, pack-years. ECOG, Eastern Cooperative Oncology Group Performance Status.

**Table 2:** Baseline characteristics and clinical outcomes of patients with bland portal vein thrombosis stratified by anticoagulation status.

	With AC (n = 30)	Without AC (n = 9)	*p*-Value
Ascites	10 (34.5%)	1 (10%)	0.228
Age	66.41 ± 1.69	65.9 ± 2.63	0.438
BMI	28.34 ± 0.95	26.98 ± 1.24	0.220
Bilirubin	22.57 ± 12	21 ± 10.33	0.358
Sodium	137.75 ± 3.99	138.5 ± 1.51	0.284
PLT	123.51 ± 65.58	113.67 ± 41.94	0.338
AFP	7408.47 ± 25.705	565.1 ± 1135.1	0.205
HbA1c	5.69 ± 1.03	5.63 ± 1.17	0.448
Albumin	29.6 ± 5.66	30.3 ± 6.58	0.382
Bleeding episodes	4 (13.8%)	-	

Abbreviations: AC, anticoagulation; AFP, alpha-fetoprotein; BMI, body mass index; HbA1c, glycated hemoglobin; PLT, platelets.

**Table 3:** Discrete time survival analysis in patients with and without anticoagulation.

Outcome ^†^	Time	Without AC	With AC	*p* (Group)	*p* (Time)	*p* (Group*Time)
Platelets	Baseline	113.0 (64.1–161.9)	121.3 (95.6–147.0)	0.761	0.060	0.091
	3 months	127.6 (77.8–177.5)	124.8 (97.7–151.9)			
	6 months	143.4 (91.5–195.4)	115.4 (87.6–143.1)			
	12 months	157.0 (103.4–210.6)	123.4 (95.4–151.4)			
Albumin	Baseline	29.6 (25.5–33.6)	29.9 (27.6–32.2)	0.884	0.631	0.316
	3 months	28.7 (23.9–33.5)	28.7 (26.1–31.3)			
	6 months	31.5 (25.9–37.1)	29.1 (26.2–32.1)			
	12 months	32.0 (26.4–37.6)	26.8 (23.9–29.7)			
INR	Baseline	1.2 (1.0–1.4)	1.2 (1.1–1.3)	0.824	0.877	0.681
	3 months	1.2 (1.0–1.4)	1.4 (1.2–1.5)			
	6 months	1.1 (0.9–1.4)	1.3 (1.1–1.4)			
	12 months	1.3 (0.9–1.6)	1.4 (1.3–1.6)			
Bilirubin	Baseline	22.9 (17.2–30.6)	23.1 (19.4–27.4)	0.973	0.046	0.020
	3 months	32.6 (23.9–44.4)	21.4 (17.4–26.2)			
	6 months	20.9 (13.4–32.7)	23.0 (18.8–28.2)			
	12 months	13.3 (6.9–25.4)	26.2 (21.4–32.1)			
AFP	Baseline	37.0 (4.1–337.0)	40.4 (11.8–138.1)	0.946	0.145	0.089
	3 months	65.0 (7.0–606.2)	34.5 (9.9–120.4)			
	6 months	37.0 (4.1–337.0)	40.4 (11.8–138.0)			
	12 months	64.9 (7.0–605.5)	34.5 (9.9–120.4)			
MELD	Baseline	8 [8; 10]	9 [8; 11]	0.535	0.552	0.837
	3 months	10 [8; 11]	10 [8; 11]			
	6 months	10 [8; 11]	10 [8; 11]			
	12 months	10 [9; 11]	11 [10; 6]			
AKI	3 months	86 [80; NA]	84 [76; 88]	<0.001	<0.001	<0.001
	6 months	70 [66; 76]	84 [75; 88]			
	12 months	83 [75; 88]	71 [67; 80]			

^†^ Values are model-based marginal means (95% CI) or medians [IQR]. bilirubin (lognormal GLMM) and AFP (Gamma GLMM) shown as geometric means on original scale; MELD and AKI (ordinal GLMM) show medians based on cumulative probabilities. Type III Wald χ^2^ *p*-values. AC—anticoagulation.

## Data Availability

The data that support the findings of this study are available from the corresponding author upon reasonable request.

## Supplementary Materials

The following supporting information can be downloaded at https://www.mdpi.com/article/10.3390/cancers18132148/s1, Figure S1: Comparison of platelet counts between patients without portal vein thrombosis (PVT) and those who developed PVT during follow-up.
