# Supplementary figures and images for: Natural History of Treated and Untreated Bland Portal Vein Thrombosis in Patients with Hepatocellular Carcinoma
Source: Cancers (Basel). 2026 Jul 3;18(13):2148. doi: 10.3390/cancers18132148 (PMC13359517; doi:10.3390/cancers18132148)

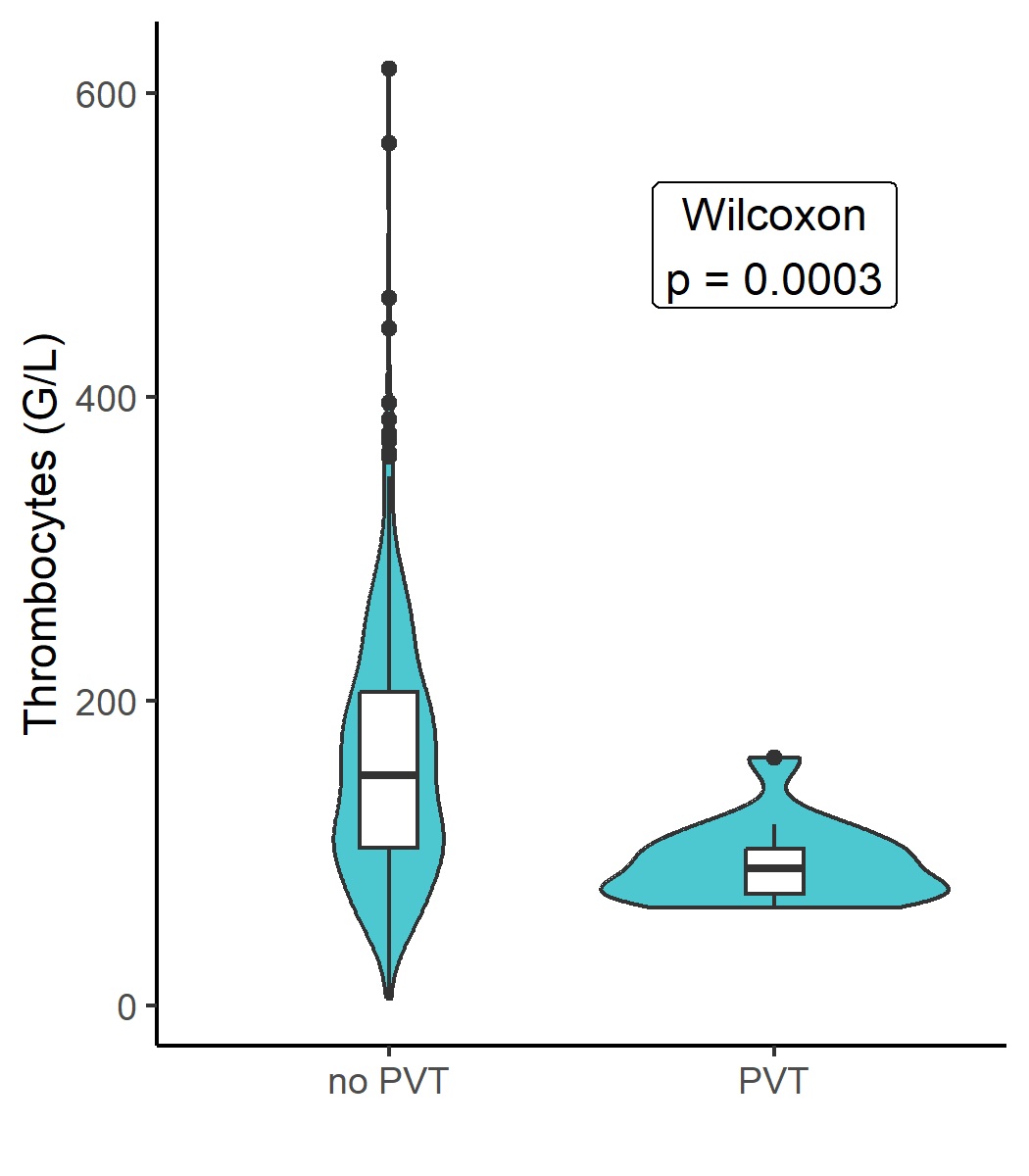

Supplement: Supplementary file 1 [file cancers-18-02148-s001.zip › Figure S1.jpg]
